# Prebiotic chemistry and human intervention

**DOI:** 10.1038/s41467-018-07219-5

**Published:** 2018-12-12

**Authors:** Clemens Richert

**Affiliations:** Institute of Organic Chemistry, University of Stuttgart, 70569 Stuttgart, Germany

## Abstract

Experimentalists in the field of prebiotic chemistry strive to re-enact what may have happened when life arose from inanimate material. How often human intervention was needed to obtain a specific result in their studies is worth reporting.

## Introduction

When Diego Maradona was asked about having used his hand to score a goal in the quarter-finals of the 1986 soccer World Cup, he initially claimed that there had been divine intervention, and the term “Hand of God Goal” was coined.—There had been manual intervention, and there had been an understandable interest of the player not to admit it.—Organic chemists, if not all experimentalists in the field of prebiotic chemistry, are faced with a similar dilemma. We do our best to perform experiments that we believe re-enact possible steps of prebiotic evolution, but we know that we need to intervene manually to obtain meaningful results. Simply mixing chemicals and watching for a living system to appear from the broth seems unreasonable to me. This approach has never worked, and it is not expected to work, at least not if one is limited to the lifetime of a human, let alone the duration of a funding period or a Ph.D. thesis. So, what is a reasonable level of intervention by the experimentalist in prebiotic chemistry, and what are “plausible prebiotic conditions” in this context?

For experimental scientists, there is a need to start from defined conditions, and results can only be trusted, if they are reproducible. So, there is little doubt that chemists will want to start from known quantities of pure chemicals. Such a pure-chemicals scenario is unrealistic prebiotically (organic compounds produced from simpler starting materials on planets are usually found as mixtures of structurally related molecular entities), but necessary.

Further, the ideal experiment does not involve any human intervention. A reaction or a reaction network is allowed to unfold, and the sample is only broken up when the experiment has been finished. Perhaps, samples are drawn, as in the famous Miller experiment^1,2^, but there is no addition of new chemicals or an artificial change in conditions.

This type of single-run experiment has worked well for simple processes, such as the formation of sugars in a formose reaction^3^, the oligomerization of activated nucleotides to give short RNA strands^4^, or the Miller volcanic spark experiment that produces simple biomolecules mentioned above^1^. For experiments aimed at demonstrating chemically more complex processes, such as multistep syntheses mimicking biochemical pathways or genetic replication, repeated interventions by the experimentalist have been necessary. Each step needs a specific chemical environment or set of conditions to occur in high yield. For example, an elimination reaction needs other conditions than an addition reaction, and assuming that both will occur simultaneously in the same solution is unrealistic.

In the cell, the individual steps of a biosynthetic pathway are usually catalyzed by different enzymes. Each enzyme creates a specific microenvironment for a reaction in its active site. For potentially prebiotic, enzyme-free multistep syntheses, a chemical work-up at the end of a reaction is often required, involving steps such as precipitation, crystallization or other forms of handling and purification, and an often drastic change in chemical conditions from one synthetic transformation to the next. Understandably, this has drawn the ire of those who feel that no or only minimal intervention is allowed for a process to be called prebiotically plausible. After all, it is not easy to see what replaced the flasks, pipettes and stir bars of a chemistry lab during prebiotic evolution, let alone the hands of the chemist who performed the manipulations. (And yes, most of us are not comfortable with the idea of divine intervention in this context.)

It is not easy to compute a score for how prebiotically plausible an experiments is. Often, if a reagent has already been found somewhere in the geosphere, it is considered plausible, and if it was taken from the catalog of a chemical supplier, it is not. Is this reasonable? Further, how likely it is that a series of transformations occurred that require vastly different conditions in a specific sequence, is not easy to gauge, even for the specialist. Still, this parameter may be more important than the source of the chemicals. Temperature changes are plausible, based on seasonal changes and day/night cycles, hydration and dehydration are plausible, based on rainfall, tides and other processes in the hydrosphere, but going well beyond a range of, say, −20 to 100 °C for aqueous media or assuming that arctic conditions change to volcanic conditions within hours or days seems unreasonable to me. For processes requiring base pairing outside the active site of enzymes, temperatures above 85°C seem unrealistic, based on the known stabilities of duplexes. For base pairing involving single nucleotides, as in genetic copying, we do not see significant template effects above body temperature (37 °C) in primer extension assays, suggesting that these temperatures are too strongly denaturing. Also, how concentrated specific organic compounds may have become in one specific location on the early Earth, based on temperature gradients and diffusion, is unclear. Not every geochemist may agree with specific assumptions, and as a consequence of such issues, scholarly debates over what is prebiotically plausible and what is not, have been controversial.

Plausibility is important. So, perhaps it is time to think about ways out of the “Hand of God” dilemma. Three things come to mind.

First of all, I feel is it reasonable to report the number of manual interventions during an assay explicitly. This number can be quite high, as in the case of enzyme-free replication from activated nucleotides reported by us, where washing and deprotection steps were necessary to be able to measure the level of misincorporation of nucleotides mass spectrometrically^5^. It can also be high for multistep syntheses, mimicking entire biochemical pathways^6,7^. Understandably so, as self-organizing biochemical cycles are difficult to demonstrate experimentally^8^. Usually, one tries to keep the number of steps in the single digit range. When it becomes unavoidable to intervene as experimentalist, just state the number of discontinuities in the experimental conditions or human interventions!

Secondly, one may want to state more explicitly what prebiotic scenario a specific experiment is believed to address. This will not solve the “Hand of God dilemma”, but it may allow the reader to gauge what geochemical conditions were assumed when the experiment was planned. Is the experiment focused on the formation of simple biomolecules from inorganic precursors, is it an experiment aimed at understanding the reactivity of such biomolecules, or is it an experiment tackling multistep processes, such as synthetic pathways or genetic replication? What is the presumed stage of prebiotic evolution that is the backdrop of the experiment performed?

Thirdly, it makes sense to reduce the number of interventions required for an experiment, e.g. by employing non-invasive spectroscopic techniques and building on known reaction networks that produce multiple biomolecules in one solution^9,10^. The power of multidimensional NMR spectroscopy, combined with modern mass spectrometry, allows one to monitor multiple biochemical species in one solution over extended periods of time. The analysis of the data can be a time-consuming jigsaw puzzle to solve, but this is well worth the effort, given that this can minimize the number of interventions.

A final word of caution. Life is a non-equilibrium phenomenon. It requires an energy source that drives its reactions. Assuming that simple heating/cooling cycles could have driven the formation of functional biomacromolecules that were then able to harness the energy emitted by the sun via photosynthesis, seems unrealistic to me. Achieving the level of specificity required to successfully operate a protocell with genetic apparatus, metabolism, and cell division under strongly denaturing conditions is not easy, certainly when it comes to enzyme-free replication relying on the intrinsic specificity of small molecule interactions. So, the periodic addition of a chemical condensing agent may be unavoidable to drive biochemical reactions that are endergonic, even in “minimal intervention” experiments. Without the chemical activation, equilibrium (death) sets in. So, some level of human intervention may always be required for complex, multistep processes. After all, what the dominant activation agent was before enzymes began to use ATP will remain an enigma to many of us for the foreseeable future.
